# Responses to Concerning Posts on Social Media and Their Implications for Suicide Prevention Training for Military Veterans: Qualitative Study

**DOI:** 10.2196/22076

**Published:** 2020-10-30

**Authors:** Alan R Teo, Wynn Strange, Ricky Bui, Steven K Dobscha, Sarah S Ono

**Affiliations:** 1 Health Services Research and Development Center to Improve Veteran Involvement in Care VA Portland Health Care System Portland, OR United States; 2 Department of Psychiatry Oregon Health and Science University Portland, OR United States; 3 School of Public Health Oregon Health and Science University and Portland State University Portland, OR United States; 4 Veterans Rural Health Resource Center–Portland Veterans Health Administration Office of Rural Health Portland, OR United States; 5 Department of Family Medicine Oregon Health and Science University Portland, OR United States

**Keywords:** concerning post, social media, suicide prevention, gatekeeper training, military veterans

## Abstract

**Background:**

A “concerning post” is a display of a user’s emotional crisis on a social media platform. A better understanding of concerning posts is relevant to suicide prevention, but little is known about social media users’ attitudes and responses to concerning posts. Military veterans in the United States are disproportionately affected by suicide, often use social media, and may have exposure to individuals with elevated suicide risk via concerning posts.

**Objective:**

The objective of the study was (1) to obtain insight into whether and how US military veterans respond to members of their social network on social media (ie, “friends”) who are experiencing substantial emotional distress, and (2) to identify potential interventions that could assist in users’ response to concerning posts.

**Methods:**

We recruited veterans through Facebook and conducted semistructured interviews with 30 participants between June and December 2017. We used a summary template for rapid analysis of each interview, followed by double-coding using a codebook based on topic domains from the interview guide. Members of the research team met regularly to discuss emerging patterns in the data, generate themes, and select representative quotes for inclusion in the manuscript.

**Results:**

Veterans were reluctant to disclose emotional and health issues on Facebook, but they were open to reaching out to others’ concerning posts. There was a complex calculus underlying whether and how veterans responded to a concerning post, which involved considering (1) physical proximity to the person posting, (2) relationship closeness, (3) existing responses to the post, and (4) ability to maintain contact with the person. Veterans desired additional training, backed by community-based veteran organizations, in how to respond to concerning posts from peers.

**Conclusions:**

There is a need to incorporate features that will help veterans effectively respond to concerning posts from peers into suicide prevention training and to expand access for veterans to such training.

## Introduction

As social media has become embedded into our daily lives, researchers have taken on the task of unpacking its myriad implications for our health. Studies of social media have often focused on examining psychosocial harms from exposure to social media, such as loneliness [1], negative social comparisons [2], and worsening of mental health status [3]. Perhaps because of negative public and scientific perceptions of social media, less attention has been paid to identifying ways that social media could be used to improve mental health [4]. In reality, both positive and negative consequences of social media coexist and are influenced by how individuals—and society collectively—interact using social media.

An especially delicate but important health concern in the social media landscape is suicide prevention. Suicidal individuals are more likely than nonsuicidal individuals to spend time online [5]. Expressions of suicidal ideation on social media have been widely reported and, at times, are followed by suicidal behavior [6,7]. Disclosures of emotional distress on social media reflect real-life concerns, with studies showing links between depressive language in Facebook posts and self-reported symptoms of depression and medical record–confirmed diagnoses of depression [8,9]. Even more visible to the public are media reports about suicide, which are relatively common on social media compared with reports of deaths from other causes [10]. Analysis of Twitter data has found that large reactions to this reporting, measured through increased mentions after the death of a celebrity by suicide, are linked to subsequent increases in deaths by suicide [11].

Amidst this contemporary reality, the notion of a “concerning post” has emerged, which we define as a display of a user’s emotional crisis or other indication of an acute mental health concern on a social media platform. Little is known about individuals’ attitudes and responses to concerning posts. Among the available studies, results have conflicted at times. For instance, one study found that youth lacked confidence in how to respond to a concerning post [12], while another study found that sharing emotional distress activated supportive responses [13].

While much of the research in this area has focused on youth, military veterans are another key target population for suicide interventions. Veterans in the United States are disproportionately affected by suicide. Their rate of suicide is 50% (1.5 times) higher than that of nonveterans, according to the most recent available data [14]. Suicide exposure (knowing someone who has died by suicide) is also very common and may occur via social media; an estimated 57% of veterans [15] and 65% of National Guard personnel [16] have been exposed to suicide. Social media plays an important role in maintaining veterans’ social networks, with one contributor being the physical separation from family and friends that occurs during deployment or active duty, as well as separation from their servicemember peers after leaving the military. Social media serves to sustain connection and communication over time and in particular through periods of transition [17].

Using data from interviews with veterans who use social media, our objectives in this study were (1) to obtain insight into whether and how veterans respond to “friends” and family members on social media who are experiencing substantial emotional distress, and (2) to identify potential interventions that could assist veterans in responding to concerning posts.

## Methods

### Setting and Sample

This research was conducted as part of a mixed-methods study focused on understanding opportunities to use social media platforms, particularly Facebook, to reach and provide support to veterans at risk of psychiatric problems. We interviewed Operation Enduring Freedom, Operation Iraqi Freedom, and Operation New Dawn veterans with a service separation date after October 1, 2001, who were in the metropolitan area of Portland, Oregon, United States. Thirty participants were interviewed—a number chosen based on the literature, feasibility, and our research team’s experience reaching thematic saturation with such a sample size [18,19].

### Data Collection

Participants who had responded to Facebook advertisements and completed an online survey were eligible for participation in the current study. The survey that was administered has been previously described [20,21]; in brief, it included items on sociodemographic characteristics, frequency of social contact, use of social media platforms, screening for psychiatric problems and health service utilization, and interest in potential online interventions for social support and suicide prevention. Among participants who used Facebook daily, we purposively sampled veterans that varied in the extent to which they used the Department of Veterans Affairs for their health care and varied in their interest in learning more about suicide prevention.

Fifty individuals were contacted to participate, and 30 of them completed an approximately 40- to 60-minute interview between June and December 2017. Twenty-three interviews were completed in person; the remaining seven interviews were conducted over the phone, which allowed for participation by individuals who could not present in person to participate in the study. Two research team members (HM and AT) conducted the interviews using a semistructured interview guide (Multimedia Appendix 1). Questions were organized into three primary domains: (1) participants’ social media use and preferences, (2) discussion of health issues on social media, and (3) suicide prevention in a social media context (including experiences with concerning posts and preferences related to suicide prevention training). Interviews were audio recorded and transcribed.

### Data Analysis

Survey responses were used for participants’ descriptive characteristics. For qualitative analysis, we first created a summary template for rapid analysis of each interview [22,23]. We then created a codebook based on topic domains from the semistructured interview guide and informed by notes from the summary templates. Two research assistants (RB and WS) coded the interviews using qualitative analysis software (Atlas.ti; ATLAS.ti Scientific Software Development GmbH) for data management. As the primary coder, RB met weekly with the lead investigator (AT) to discuss general impressions and emergent themes. As the secondary coder, WS reviewed the primary coding, adding any codes that may not have been applied, with the goal of ensuring robust coding. AT adjudicated coding disputes as needed. Members of the research team (AT, RB, WS, SD, and SO) met regularly to discuss emerging patterns in the data, generate themes, and select representative quotes for inclusion in the manuscript. We assigned pseudonyms to the participants to maintain confidentiality.

## Results

### Principal Findings

A summary of participants’ characteristics is presented in Table 1. The 30 participants (23 men, 7 women) came from all military branches, with 3 participants serving in two branches over their careers. The average age of participants was 41 years (range 24-64 years), 73% (22/30) were White, 60% (18/30) were married, and 57% (17/30) had a college or postgraduate degree. In terms of their mental health, 5 of 30 (17%) participants received a score of 3 or greater on the Patient Health Questionnaire-2, which is indicative of a positive screen for major depressive disorder [24]; 15 of 30 (50%) participants had considered suicide, and 5 of 30 (17%) participants had previously attempted suicide. While 90% (27/30) of participants used Facebook several times a day, the majority never or rarely used social media for sharing information about their health (23/30, 77%), symptoms such as mood swings, depression, anxiety, or sleep problems (21/30, 70%), or thoughts about suicide or self-harm (28/30, 93%).

Veterans were reluctant to disclose emotional and health issues on social media but were open to reaching out in response to others’ concerning posts. Interviews revealed multiple reasons for veterans’ reluctance to share negative emotional states or information about their health. First, Facebook was viewed as a venue for positive self-presentation; thus, they were disinclined to use it as a place to “air dirty laundry.” Second, when veterans did need support, they usually preferred to obtain it from other avenues in the “real world” that involve “person-to-person interaction,” or they preferred to be self-reliant. Also, veterans believed that sharing personal health information was “risky” to their privacy. Lastly, participants noted that Facebook friends were not necessarily “real friends.”

While most veterans were cautious about disclosing their own health issues, a number of veterans were understanding of others who chose to post about health issues. One participant summarized this distinction:

I think for some people they find that they can post about their bad day, and they get a bunch of people “like” it, a bunch of people put the sad face on, a bunch of people say, “You’re amazing. You’re brilliant. You go girl.” That kind of thing. I’ve definitely seen a ton of people do that, but at the same time I would never do that. And it’s not like I don’t want support, but I feel like I would be negatively judged for that.

Oscar

Further, many veterans were open to responding when others disclosed personal information in a post, particularly emotional struggles or suicidality.

I’ve seen other friends do it, and I’ve come to the aid of other friends because they have shared it on Facebook out in the open just to their timeline or whatever. I’ve also helped friends who have reached out to me personally and [sent] Facebook messages and said, “I’m having this crisis. I can use some words of encouragement or some words of advice or some help just in general.”

Bryan

Participants identified a post as concerning in several ways. Most obviously, this was direct language expressing emotional distress, particularly when the distressed person had a known mental health condition (eg, post-traumatic stress disorder or an anxiety disorder), when the person was acting out of character (eg, getting rid of their possessions), or if the person had one or more recent psychosocial stressor, such as divorce/breakup, death of a loved one, or loss of a job. Several veterans also expressed a feeling of concern without an identifiable cause, noting, for instance, “[The post] just seems off” [Seth]. Veterans occasionally did not interpret a post as concerning if they felt the post was “venting” [Natasha] or that the person was seeking attention.

There was a complex calculus behind whether and how individuals responded to a concerning post. Once veterans had determined a post to be concerning, they wanted to be there for a peer in distress, but they did not always know if and how they should respond. Before they reached out, veterans went through a series of considerations. Elements of this complex calculus included (1) physical proximity, (2) relationship closeness, (3) existing responses to the post, and (4) ability to follow up with the person in crisis. While the weight given to each consideration varied across situations and circumstances, each factor influenced decisions related to whether veterans responded, how they chose to reach out, and how urgently and directly they responded.

When a friend posted something on Facebook that appeared to indicate suicidal ideation, participants reported that the preferred response was to attempt to make direct, one-on-one contact to talk with the friend. This direct contact could be through Facebook itself (using a private message), email, a phone call or text, or meeting up in person. Multiple methods of contact could be combined, typically with the goal of having the online contacts lead to an in-person contact.

**Table 1:** Summary of participants’ demographic, clinical, and social media use characteristics (N=30).

Characteristics				Mean (range)	n (%)
**Demographic characteristics**					
	Age (years)			41 (24-64)	
	Gender				
		Male			23 (77)
		Female			7 (23)
	Race/ethnicity				
		White			22 (73)
		Hispanic			3 (10)
		American Indian/Alaska Native			2 (7)
		Asian			3 (10)
	Educational attainment				
		High school or high school equivalency certificate			1 (3)
		Some college			9 (30)
		Technical or vocational school			3 (10)
		College graduate			11 (37)
		Postgraduate			6 (20)
	Marital status				
		Single, never married			7 (18)
		Divorced			4 (13)
		Separated			1 (3)
		Married			18 (60)
	Military branch^a^				
		Army			11 (37)
		Marine Corps			9 (30)
		Navy			6 (20)
		Air Force			5 (17)
		Coast Guard			2 (7)
**Clinical characteristics**					
	Patient Health Questionnaire-2 score			1.5 (0-4)	
		0			11 (37)
		1			2 (7)
		2			12 (40)
		3			2 (7)
		4			3 (10)
	History of serious suicidal ideation^b^				
		Yes			15 (50)
		No			15 (50)
	Number of prior suicide attempts^c^				
		0			25 (83)
		1			2 (7)
		2			2 (7)
		3			0 (0)
		4			1 (3)
**Social media use**					
	How often do you visit or use Facebook?				
		Several times a day			27 (90)
		Once a day			2 (7)
		A few times a week			1 (3)
	How often do you use any social media website to do the following:				
		Share symptoms such as mood swings, depression, anxiety, or sleep problems?			
			Never		14 (47)
			Rarely		7 (18)
			Sometimes		4 (13)
			Usually		4 (13)
			Always		1 (3)
		Share information related to your health?			
			Never		10 (33)
			Rarely		13 (43)
			Sometimes		5 (17)
			Usually		2 (7)
			Always		0 (0)
		Share thoughts about suicide or hurting yourself in some way?			
			Never		26 (87)
			Rarely		2 (7)
			Sometimes		2 (7)
			Usually		0 (0)
			Always		0 (0)

^a^Total is greater than 100% because three participants served in two branches.

^b^Survey item asked, “Have you ever seriously considered attempting suicide at some point in your life?”

^c^Survey item asked, “How many times in your life have you attempted suicide?”

### Physical Proximity

When veterans saw a concerning post on Facebook, one of the first considerations for how they chose to respond was their physical proximity to the individual in distress. If the friend was within a reasonable travel distance, veterans often indicated it was optimal to visit them or call immediately to arrange an in-person meetup as soon as possible. If a friend expressed loneliness or concerning thoughts, participants felt responding through Facebook alone was insufficient. By meeting up in person, veterans felt that they could “get them out and let them know that there are still people for them” [Jamie] and “read more in a person by watching their body language” [Robert]. If the friend was not in close proximity and the level of concern was high, some veterans noted that they might try to get a next-of-kin or other person who was physically closer to check in on the friend.

Depends on physical proximity, if it’s somebody within driving distance, within a few hours I’ll be at their house banging on the fucking door. If not, there’s going to be phone calls and emails and text messages and that’s the way I would react.

Alyssa

### Relationship Closeness

Veterans varied in their responsiveness to concerning posts depending on the closeness of their relationship with the person in crisis. For instance, if the concerning post was from an acquaintance or someone that was on the periphery of their life, they were more likely to respond in a less direct or less immediate fashion.

I'll send them the phone number for the veteran's suicide hotline, that’s really about as far as I'm willing to go unless I know the person.

Andrea

If I don’t know them personally, I try to give an encouraging thought.

Natasha

For a close relationship, efforts to respond were more direct and more intensive. One veteran said that in the case of a close friend or family member, he would respond as follows:

I probably wouldn’t even be on social media in that case. I would’ve just gone to the person and, you know, physically gone to see them.

Conrad

For relationships that fall somewhere between acquaintance and close friend or family, participants tended to communicate their availability but ultimately left the onus of further connection on the person in crisis. These efforts were often made under the assumption that there was someone closer who could help the person in crisis but also acknowledged that they were unsure of this and were thus willing to provide support if needed.

…you can reach out to them and just give them a little love and then you instant message him and say, “Here’s my phone number and call me if you need to.”

Jerry

### Existing Responses to the Post

A third consideration in the response calculus related to the interactions and activity in response to a concerning post, including “likes,” comments, and shares that were visible to others. Often, the timing of the post relative to when it was being seen was a factor. Veterans stressed the importance of timing for two reasons: first, the circumstances surrounding the crisis may have changed, and second, others may have already intervened.

I’ve seen it a couple times or I’ve been, for lack of better term, late to the conversation and you’ve seen other people comment before that and someone said, “Hey, I’ve talked to ‘em.”

Neal

Recognizing that timing of a post and actions by others might have resulted in resolution by the time they saw a concerning post, participants also described assessing if there had already been direct and supportive responses to the friend when deciding if they should respond.

I’m usually not the first person to come along by it, so usually I’ll check the comment section and see what’s being done. Typically, someone will comment and then they’ll say: I’ve got ahold of them, or got them talking.

Richard

And after somebody posts, there's some likes and dislikes and maybe a “hey, what's up, call me.” I imagine in those situations there's four or five personal conversations going on with that person. And then they decide who is understanding the situation, talk with them.

Yolanda

### Ability to Follow Up

Some veterans emphasized both the need to initially reach out and the importance of following up with a friend in crisis. Veterans who discussed the importance of follow-up considered whether they had the capacity to continue to maintain contact and actively provide support.

One veteran responded that she had a friend who posted on Facebook “reaching out about how lonely they were.” She went on to say the following:

…they’re obviously looking for that public acknowledgment, so, I did that. And kept it… you know, more… general, I guess, and supportive, and then, messaged them more. You know, specifically relating to it and then scheduling a follow-up visit with them.

Jamie

She let the friend know they weren’t alone and scheduled a time to meet up with them.

…*it was the next day or something. Just for a quick coffee date, you know, just to get them out and let them know that there are still people for them.* [Jamie]

…*you don’t just listen and walk away. You still have to follow-up with them and keep talking with them. It might not even be someone that you actually really know, but if you come across someone in that state of mind, it’s usually kind of obvious that something’s not quite right so don’t ignore them.* [Bryan]

### Suicide Prevention Training

Veterans desired suicide prevention training that would prepare them to assist others in emotional crisis. The suggestion for training on how to respond to concerning posts, specifically if suicidal ideation was suspected or indicated, was raised by participants as well as asked about by the interviewer. Veterans indicated a desire for training in suicide prevention skills to help a peer in crisis. A common reason they cited for wanting training was a sense of being unprepared to assist others in crisis or even worry about exacerbating the situation.

I have no idea what to say but just trying to make myself available, trying to say as little damaging stuff as I can… It’s mostly that I have no idea what should I do to help? …I got pretty much nothing else.

Alyssa

I don’t know how to sometimes get the point across to them without sounding judgmental.

Lynn

#### Training as an Ongoing Process

Even veterans who indicated having prior suicide prevention training, often during military service, felt that the training was inadequate or insufficient. This seemed especially vital for veterans who were interested in helping others but worried about how to initiate conversations with more socially distant individuals.

Would I want more? Yes. Just to let me know I’ve got the basics down. I’m not a licensed mental health professional so I can’t go about helping them solve their problems, but I can get [someone in crisis] in the right direction.

Edward

I’d like to recognize more warning signs, and I would like to have some conversation items that I could take as far as how to talk about it, or how to bring it up.

Lewis

#### Preferred Features for Suicide Prevention Training

Veterans identified two features of suicide prevention training that would make it especially appealing. First, training sponsored or delivered by community-based veteran organizations was believed to “carry more weight” [Neal] and be “a good source of credibility” [Edward]. Many participants who followed at least one veteran-centric Facebook page or group (eg, Dysfunctional Veterans, Lift for the 22, Gulf War Veterans, etc) felt these groups could act as trusted sponsors of suicide prevention training. One veteran described the kind of support he’s encountered in one such private Facebook group:

…every 22nd of every month, we do a check in. We’ll post a thing, check in with our veteran friends because it comes out that there’s 22 vets that commit suicide every day. And we started a program called “22 to None,” so we support each other. I’ve made a couple friends with some other vets, and other Navy vets. And, one guy… he told me, gave me his number, he says you ever start feeling really bad, give me a call. He was a trained counselor as well.

Keith

Second, veterans were most willing to receive training targeted at helping their veteran peers. Their shared experiences, military background, and camaraderie as a result of service made a difference in their level of comfort and trust when talking about the topic of suicide with other veterans.

It’s very easy for me to have a relationship or an exchange of information between another person who’s in the service. It’s easier than it is for me to sometimes talk with my wife, or members of my family because they don’t have that background like we have—those who’ve served.

Meghan

Have you ever had two different people tell you the same piece of information? One is Bob who you don’t really know particularly well, but the other one is somebody who is close to you—[you] know and respect—they repeat that same information, you place a lot more weight on it. With somebody who you’ve had a lot of those shared experiences with, I may not know them particularly well, but they’ve been in the same places I have, they’ve faced the same problems I’ve had, and if they say that this is helpful, I place a lot more weight on it.

Alyssa

## Discussion

### Principal Findings

In the daily course of their lives, veterans are likely to encounter others in emotional crisis, and this can manifest through concerning posts on social media platforms such as Facebook. When presented with a concerning post, veterans go through a complex calculus—involving an assessment of physical proximity, relationship closeness, activity on the post, and ability to follow-up—which informs the extent and manner in which they reach out to provide support. The order of the complex calculus and the weight of each element varies in each situation. Veterans are often inclined to help their peers in some way, but they often feel unprepared as to how to respond to a concerning post. Given these circumstances, veterans desire suicide prevention training that extends beyond what they received during military service. They seek additional training that is germane to responding to concerning posts on social media, backed by community-based veteran organizations, and tailored to providing assistance to their veteran peers.

### Links to Existing Research on Social Media and Suicide Prevention

Our finding that veterans are thoughtful and potentially motivated to respond positively to concerning posts suggests a key exception to prior research on displays of negative emotions on social media. Experimental and observational studies have shown that emotions are transmitted through social media posts [25,26]. Individuals who frequently post on social media are more likely to post about health issues and to have a diagnosis of depression [27]. Moreover, early research on Facebook posting suggested that depressed individuals see the platform as an appealing venue for self-disclosure, but their posts are often met with undesirable responses due to the negativity of the posts [28]. It may be that military culture around altruism and stewardship toward veteran peers (eg, taking care of “our own,” leaving no one behind, etc) [29] contribute to the positive intentions we found toward responding to concerning posts. Our findings also comport with research showing that individuals often fear personal expressions of vulnerability but tend to react more positively to those who express it [30].

### Training Models Applicable to Social Media–Based Suicide Prevention Training

Our findings complement the use of two education and training intervention models in the literature: gatekeeper training and bystander education. Both gatekeeper training and bystander education train individuals to recognize the signs of crisis and develop skills to triage such a situation and assist with accessing subsequent professional assistance [31,32].

Gatekeeper training has been specifically designed to support “upstream” suicide prevention. Reviews have found evidence of gatekeeper training’s ability to increase knowledge and foster self-efficacy and other beliefs adaptive to suicide prevention [32]. In a recently published pilot intervention, a gatekeeper training approach helped prepare adults to support American Indian and Alaska Native youth who post or view concerning messages on social media [33]. Bystander interventions, also called bystander education, have been primarily used for prevention of sexual violence, and they emphasize a five-step model: (1) awareness of an issue that requires intervention; (2) identifying when the bystander should intervene; (3) deciding to take responsibility; (4) deciding how to help; and (5) taking action [31]. Education about all five steps is meant to increase the likelihood of a bystander taking action at multiple time points. Both of these educational approaches would seem highly applicable to craft training for veterans and others in how to identify and respond to concerning posts. Gatekeeper training and bystander education might be strengthened if future research identifies how expressions of vulnerability and peer support could normalize help-seeking and dialogue about suicide.

### Limitations

Our findings are constrained by several study limitations. Participants were recruited from a sample of locally recruited veterans from Facebook, and thus our findings may not be entirely generalizable to veterans nationally, individuals less inclined to participate in research or to use Facebook, or individuals who have experiences on other social media platforms. The last constraint is particularly relevant given rapid shifts in the social media landscape. There are other facets of Facebook posts, such as posts within private Facebook groups or automatic identification of concerning posts using machine learning models [34], that were beyond the scope of this study but merit closer investigation as part of a larger social media suicide prevention strategy. Finally, as with all qualitative work, we took participants on their word that they accurately represented themselves and their experiences.

### Conclusions

Social media is an important venue through which many connect with their social network. One consequence of this, particularly among veterans who are disproportionately affected by and exposed to suicide, is encountering concerning posts. Our findings call for expanding access to ongoing suicide prevention training opportunities—in partnership with community-based veteran organizations—and incorporating features into existing suicide prevention training programs that will help veterans in their response to concerning posts from their veteran peers.

## Appendix

Multimedia Appendix 1 Interview Guide.

## References

[1] Primack BA, Shensa A, Sidani JE, Whaite EO, Lin LY, Rosen D, Colditz JB, Radovic A, Miller E (2017). Social Media Use and Perceived Social Isolation Among Young Adults in the U.S. Am J Prev Med.

[2] Verduyn P, Ybarra O, Résibois M, Jonides J, Kross E (2017). Do Social Network Sites Enhance or Undermine Subjective Well-Being? A Critical Review. Social Issues and Policy Review.

[3] Shakya HB, Christakis NA (2017). Association of Facebook Use With Compromised Well-Being: A Longitudinal Study. Am J Epidemiol.

[4] Pagoto S, Waring ME, May CN, Ding EY, Kunz WH, Hayes R, Oleski JL (2016). Adapting Behavioral Interventions for Social Media Delivery. J Med Internet Res.

[5] Harris KM, Mclean JP, Sheffield J (2014). Suicidal and Online: How Do Online Behaviors Inform Us of This High-Risk Population?. Death Studies.

[6] Fu K, Cheng Q, Wong PWC, Yip PSF (2013). Responses to a self-presented suicide attempt in social media: a social network analysis. Crisis.

[7] Ruder TD, Hatch GM, Ampanozi G, Thali MJ, Fischer N (2011). Suicide announcement on Facebook. Crisis.

[8] Moreno MA, Christakis DA, Egan KG, Jelenchick LA, Cox E, Young H, Villiard H, Becker T (2012). A pilot evaluation of associations between displayed depression references on Facebook and self-reported depression using a clinical scale. J Behav Health Serv Res.

[9] Eichstaedt JC, Smith RJ, Merchant RM, Ungar LH, Crutchley P, Preoţiuc-Pietro D, Asch DA, Schwartz HA (2018). Facebook language predicts depression in medical records. Proc Natl Acad Sci U S A.

[10] Scourfield J, Colombo G, Burnap P, Evans R, Jacob N, Williams M, Caul S (2019). The Number and Characteristics of Newspaper and Twitter Reports on Suicides and Road Traffic Deaths in Young People. Arch Suicide Res.

[11] Ueda M, Mori K, Matsubayashi T, Sawada Y (2017). Tweeting celebrity suicides: Users' reaction to prominent suicide deaths on Twitter and subsequent increases in actual suicides. Soc Sci Med.

[12] Gritton J, Rushing S, Stephens D, Ghost Dog T, Kerr B, Moreno M (2017). Responding to concerning posts on social media: Insights and solutions from American Indian and Alaska Native youth. Am Indian Alsk Native Ment Health Res.

[13] Park J, Lee DS, Shablack H, Verduyn P, Deldin P, Ybarra O, Jonides J, Kross E (2016). When perceptions defy reality: The relationships between depression and actual and perceived Facebook social support. J Affect Disord.

[14] Office of Mental Health and Suicide Prevention (2019). 2019 National Veteran Suicide Prevention Annual Report Internet. US Department of Veterans Affairs.

[15] Hom MA, Podlogar MC, Stanley IH, Joiner TE (2017). Ethical Issues and Practical Challenges in Suicide Research. Crisis.

[16] Bryan CJ, Cerel J, Bryan AO (2017). Exposure to suicide is associated with increased risk for suicidal thoughts and behaviors among National Guard military personnel. Comprehensive Psychiatry.

[17] Mikal JP, Rice RE, Abeyta A, DeVilbiss J (2013). Transition, stress and computer-mediated social support. Computers in Human Behavior.

[18] Mason M (2010). Sample Size and Saturation in PhD Studies Using Qualitative Interviews. Forum: Qualitative Social Research.

[19] Charmaz K (2006). Constructing Grounded Theory: A Practical Guide Through Qualitative Analysis.

[20] Teo AR, Chan BK, Saha S, Nicolaidis C (2019). Frequency of social contact in-person vs. on Facebook: An examination of associations with psychiatric symptoms in military veterans. J Affect Disord.

[21] Teo AR, Liebow SB, Chan B, Dobscha SK, Graham AL (2019). Reaching Those At Risk for Psychiatric Disorders and Suicidal Ideation: Facebook Advertisements to Recruit Military Veterans. JMIR Ment Health.

[22] Beebe J (2001). Rapid Assessment Process: An Introduction.

[23] Hamilton A (2013). Qualitative methods in rapid turn-around health services research Internet. US Department of Veterans Affairs.

[24] Kroenke K, Spitzer RL, Williams JBW (2003). The Patient Health Questionnaire-2: validity of a two-item depression screener. Med Care.

[25] Ferrara E, Yang Z (2015). Measuring Emotional Contagion in Social Media. PLoS ONE.

[26] Kramer ADI, Guillory JE, Hancock JT (2014). Experimental evidence of massive-scale emotional contagion through social networks. Proc Natl Acad Sci U S A.

[27] Smith RJ, Crutchley P, Schwartz HA, Ungar L, Shofer F, Padrez KA, Merchant RM (2017). Variations in Facebook Posting Patterns Across Validated Patient Health Conditions: A Prospective Cohort Study. J Med Internet Res.

[28] Forest AL, Wood JV (2012). When social networking is not working: individuals with low self-esteem recognize but do not reap the benefits of self-disclosure on Facebook. Psychol Sci.

[29] Greden Jf, Valenstein M, Spinner J, Blow A, Gorman La, Dalack Gw, Marcus S, Kees M (2010). Buddy-to-Buddy, a citizen soldier peer support program to counteract stigma, PTSD, depression, and suicide. Ann N Y Acad Sci.

[30] Bruk A, Scholl SG, Bless H (2018). Beautiful mess effect: Self–other differences in evaluation of showing vulnerability. Journal of Personality and Social Psychology.

[31] Amar AF, Sutherland M, Kesler E (2012). Evaluation of a Bystander Education Program. Issues in Mental Health Nursing.

[32] Burnette Crystal, Ramchand Rajeev, Ayer Lynsay (2015). Gatekeeper Training for Suicide Prevention: A Theoretical Model and Review of the Empirical Literature. Rand Health Q.

[33] Kerr B, Stephens D, Pham D, Ghost Dog T, McCray C, Caughlan C, Gaston A, Gritton J, Jenkins M, Rushing SC, Moreno MA (2020). Assessing the Usability, Appeal, and Impact of a Web-Based Training for Adults Responding to Concerning Posts on Social Media: Pilot Suicide Prevention Study. JMIR Ment Health.

[34] Liu X, Liu X, Sun J, Yu NX, Sun B, Li Q, Zhu T (2019). Proactive Suicide Prevention Online (PSPO): Machine Identification and Crisis Management for Chinese Social Media Users With Suicidal Thoughts and Behaviors. J Med Internet Res.

