# Supplementary material for: Responses to Concerning Posts on Social Media and Their Implications for Suicide Prevention Training for Military Veterans: Qualitative Study
Source: J Med Internet Res. 2020 Oct 30;22(10):e22076. doi: 10.2196/22076 (PMC7665941; doi:10.2196/22076)
Supplement: Multimedia Appendix 1 [file jmir_v22i10e22076_app1.docx]

**Interview Guide**

**Instructions to Interviewer**

This is a semi-structured interview in multiple sections. The following conventions in the interview guide are followed:

- Text in normal format should be read more or less as written.
- Text in UNDERLINED CAPITAL LETTERS should not be read to the research participant. It contains instructions for you.
- Text in (GREEN PARENTHESES AND CAPITAL LETTERS) should be replaced with the appropriate phrase. For instance, “(SOCIAL MEDIA)” could be read as “Facebook” depending on what social media the participant uses.
- Text in **bold** and underline is for emphasis.
- Text in *[blue brackets and italics]* represents optional text to read to the research participant. This may apply depending on the answer to the prior question or if the participant needs clarification of the question.

**Section 0: Interview Introduction (5 minutes)**

**Thank you** for taking the time to meet with me today. We are asking for your opinions and input because we are **interested in learning about your views on social media as a potential tool to connect with veterans for their health care needs, and to support veterans’ mental health**. We are **eager to hear from you** today because you participated in our online survey.

Usually these interviews go for about **45 minutes**. I’ll be asking you a series of questions and I will take some **notes** to help me keep track of things. There are no wrong answers. You are the expert about your thoughts and experiences, and we want to learn from what you have to say. This is a chance for you to talk **in depth**, and I encourage you to tell me as much as you can and **use examples**. Giving examples is often a useful way to illustrate a point.

A few other things to remember:

1. We are **audio-recording** this discussion because we don’t want to miss any of your comments. We’ll use only our first names today, and there will not be any names in our reports. Your comments will be kept **confidential**.
2. Your participation is **voluntary** and you can ask to skip a question or to stop the interview at any time.
3. We will give you a **gift card** after the interview as a thank you for your participation.

**Section 1: Social Media use and preferences (5 minutes)**

First, I’d like to get a quick sense of your use of social media.

| **Core Questions** | **Notes and Probes** |
| --- | --- |
| 1. On the survey you completed, you mentioned using (LIST SOCIAL MEDIA SITES). Is that right? Are there others you use? 2. Which platform do you use the most? 3. What do spend your time doing on social media? 4. What do you find reliable or trustworthy on (SOCIAL MEDIA)? And what’s unreliable or inaccurate on SOCIAL MEDIA)? 5. In what ways is Facebook a good place for social support? 6. Most of the veterans who took the survey seemed to say they **were not interested in learning how to increase/get more support from** friends and family through Facebook.   What do you think about these results? Could you say why this might be so? Or why not? | - Facebook? Instagram? Twitter? - Snapchat? - YouTube? Pinterest? Tumblr? - Google+? YikYak? - Why? - Sharing? - Commenting? - Reading? - Posting? - Liking? - How can you tell? - Can you give me an example of a time when you did this? - Are there certain topics where you lean on social media the most? - Why not? - What does social support mean/feel like when it’s online? - Can you tell me more? - Can you provide an example of when you felt supported on Facebook? |

**Section 2: Outreach Through Social Media for Health Issues (5-10 minutes)**

Now I’d like to have you think about how health issues might come up in your social media experience.

| **Core Questions** | **Notes and Probes** |
| --- | --- |
| 1. ACCEPTABILITY: In what ways would social media be a good place to help you with health or medical questions or concerns? 2. ACCEPTABILITY: Does your opinion change if it is not just any health or medical issue but a mental health one specifically? 3. PREFERENCES: Many veterans told us on the survey that they were interested in an online service for stress tips or to talk about symptoms they are dealing with. What do you think that might look like?   IF PARTICIPANT ASKS ABOUT WHETHER THESE SERVICES ARE AT VA OR OHSU: [*We’re interested in online services that aren’t necessarily connected to either the VA or OHSU. We’re exploring different options at the moment.*] | - Why is that? - How would you feel about a person online calling themselves a “health coach,” to be the person to help you with these issues? - What would be helpful? - What would you consider using? |

**Section 3: Suicide Prevention (15-20 minutes)**

Okay, now we’re going to switch gears a little bit. I know talking about suicide can be uncomfortable, but it’s something we’ve all thought about. I’d like to get some more of your thoughts on some services or programs we might develop to help with issues like.

| **Core Questions** | **Notes and Probes** |
| --- | --- |
| 1. Most of the veterans on the survey seemed to indicate that they do not use any social media sites as a place to share having suicidal thoughts. Would it be appropriate to share this type of thing on social media? 2. ANECDOTE: Many veterans told us on the survey that they were interested in learning how to help someone if they were experiencing suicidal thoughts. Have you ever been in a situation where you wanted to help someone in crisis? 3. PREFERENCES (CONTENT): What would you want to learn in order to help a friend or family member having suicidal thoughts? 4. PREFERENCES (DELIVERY): How would you want to learn that [*information/skill*]? 5. What are your thoughts about a free online training in learning what to do if someone you knew were suicidal? 6. ACCEPTABILITY/APPROPRIATENESS: Let me tell you a little more about one possible training we are thinking about and get your reaction. We could do a training where you learn a few practical suicide prevention skills.   THE GOAL HERE IS TO FIND OUT HOW TO APPEAL TO THEM FOR AN ONLINE TRAINING – DON’T LIMIT THIS TO IMAGES AND/OR TEXT: [*What would make an online training such as this appealing to you? What would motivate you to take it?*] | - If you saw this kind of a post – one indicating suicidal thoughts – how would you respond? - Can you share an example or story? - It doesn’t have to be a story about suicidal thoughts in particular, but any time you were concerned about someone based on what they posted. What did you do? Is that different than what you wanted to do? - Any particular information? Skills? - What kinds of features would make it acceptable to you? - Length? - Who teaches it? - Who sponsors it? - What would help make you more likely to respond to a Facebook ad about this sort of online training? - Such as:   - How to ask a person if they are having suicidal thoughts   - How to persuade them to get care   - How to refer them to a professional. - What are your opinions about something like that? |

**Section 4: Health Services (3 minutes)**

Finally, I’d like to briefly ask you about VA benefits.

| **Core Questions** | **Notes and Probes** |
| --- | --- |
| 1. PREFERENCES: Many veterans on our survey said they were interested in an online service that could help them learn how to use VA benefits. Could you say more about why that is, or what they might be looking for? 2. PREFERENCES: What kinds of particular services or benefits would you like to be able to get from the VA? | - Where would you go to get it? - What are examples of info or topics you might want to know more about? - What would that look like? How would you design it? - What features should it have? |

**Section 5: Conclusion (Optional)**

I’ve asked you many questions. Are there important things you think were not asked? Or are there any other things you would like to share with us?

|  |
| --- |

**Interviewer Notes:**

|  |
| --- |
